# Tracking of plus-ends reveals microtubule functional diversity in different cell types

**DOI:** 10.1038/srep30285

**Published:** 2016-07-27

**Authors:** M. Reza Shaebani, Aravind Pasula, Albrecht Ott, Ludger Santen

**Affiliations:** 1 Department of Theoretical Physics, Saarland University, 66041 Saarbrücken, Germany; 2 Department of Experimental Physics, Saarland University, 66041 Saarbrücken, Germany

## Abstract

Many cellular processes are tightly connected to the dynamics of microtubules (MTs). While in neuronal axons MTs mainly regulate intracellular trafficking, they participate in cytoskeleton reorganization in many other eukaryotic cells, enabling the cell to efficiently adapt to changes in the environment. We show that the functional differences of MTs in different cell types and regions is reflected in the dynamic properties of MT tips. Using plus-end tracking proteins EB1 to monitor growing MT plus-ends, we show that MT dynamics and life cycle in axons of human neurons significantly differ from that of fibroblast cells. The density of plus-ends, as well as the rescue and catastrophe frequencies increase while the growth rate decreases toward the fibroblast cell margin. This results in a rather stable filamentous network structure and maintains the connection between nucleus and membrane. In contrast, plus-ends are uniformly distributed along the axons and exhibit diverse polymerization run times and spatially homogeneous rescue and catastrophe frequencies, leading to MT segments of various lengths. The probability distributions of the excursion length of polymerization and the MT length both follow nearly exponential tails, in agreement with the analytical predictions of a two-state model of MT dynamics.

Microtubules are semiflexible polymers with an intrinsic structural polarity. They represent tracks for the transport of material within the cell by means of molecular motor proteins. Active transport is essential for an efficient delivery of cargoes to specific locations through the crowded cytoplasm^1^, and several types of diseases arise due to perturbations in intracellular transport processes. The dynamic structure of microtubules (MTs) has been suggested to be beneficial for reducing jam formation and maintaining homogeneous states in bidirectional transport of molecular motors^2^. The transport efficiency may be dramatically affected by the drugs which stabilize (e.g. taxanes) or destabilize (e.g. vinca alkaloids) MT structure^3^. Besides the role of MTs in material delivery, their dynamics enables the cells to quickly remodel their cytoskeleton in response to environmental changes^4^. This leads to an efficient control of vital processes such as mitosis and cell division, motility, and morphogenesis. In cell types that benefit from the presence of MTs to adjust their morphological requirements, having a stable MT network near the cell margin is necessary, in contrast to cell types where MTs are not involved in the steady remodeling of the cell shape. In such cases, for example in neuronal axons, a more dynamic MT structure may be even more advantageous because of enhancing the transport capacity. The ability of MTs to rapidly switch between growth and shrinkage states, known as *dynamic instability*^5^, is assigned to the complex interplay between the applied stresses^6^,^7^, GTP hydrolysis^5^, and regulatory proteins including molecular motors^8^,^9^,^10^,^11^ and MT-associated proteins^11^,^12^,^13^,^14^.

In the present study, we demonstrate that the differences in MTs function in different cell types and regions is reflected in their dynamic structure. To this aim we label MT plus-ends by means of EB1 proteins and compare their motion in fibroblast and human neuronal cells. Transfection of human neurons is technically challenging, however, uncovering the details of MT dynamics in such cells is of crucial importance to diagnose, treat, or even prevent neurodegenerative disorders. MT dynamics have been studied in different nerve cells such as mouse hippocampal^15^, Aplysia^16^ and Drosophila^17^ neurons. We report, for the first time, the structural properties of MTs in human neuronal cells by culturing SH-SY5Y cells, a well documented human derived neuroblastoma cell line which differentiates to mature neurons after treatment with All-trans retinoic acid and brain-derived neurotrophic factor^18^. The SH-SY5Y cell line is widely used as an *in vitro* model to study biochemical and functional properties of neurons. We clarify the differences between axonal MT polymerization/depolymerization excursions as well as the spatial homogeneity of their plus-end tips with those of fibroblast cells.

The MT-associated proteins (MAPs) may stabilize or destabilize MTs in living cells by temporally or spatially regulating their dynamics. MAPs target MT-ends and/or walls, or the non-polymerized tubulin subunits. Among various types of MAPs, the plus-end tracking proteins (+TIPs) accumulate at growing MT plus-ends and play important roles e.g. in regulation of MT dynamics, delivery of signaling molecules, and control of MT interactions with other intracellular structures^19^,^20^,^21^,^22^. +TIPs may interact with each other and construct plus-end complexes. Particularly, the end-binding protein-1 (EB1) is frequently involved in such complex structures^23^. EB1 is a member of dynamic and enigmatic family of +TIPs, which is highly conserved from humans to yeasts and plants, and acts as an exquisite marker of dynamic MT plus-ends^24^,^25^. EB1 senses conformational changes, which occur in the MT lattice, linked to the GTPase cycle of tubulin at growing MT ends^26^. This leads to the autonomous comet-like accumulation of EB1 at the growing MTs.

In axons MTs are generally oriented, with their plus (minus) ends pointing toward the axon terminals (the soma)^17^,^27^. In contrast to many eukaryotic cells in which the minus ends of MTs are mainly anchored at the MT organizing center, MTs do not reach from soma all the way to axon terminals in neurons. Instead, there is an overlapping array of short segments of MT with a typical length scale of a few micrometers.

Here, we extract the length distribution *P*(*L*) of MT segments from the spatial distribution of the labeled plus-ends and show that the tail of *P*(*L*) decreases nearly exponentially. By means of a two-state model of MT growth and shrinkage, it is demonstrated how the steady-state length distribution depends on the phenomenological parameters: the growth and shrinkage rates and the frequency of catastrophe and rescue events, i.e. switching between growth and shrinkage states and vice versa.

## Methods

### Cell culture and differentiation

SH-SY5Y cells were cultured in growth medium containing Dulbecco’s Modified Eagle Medium (DMEM; Gibco) supplemented with 10% heat-inactivated Fetal Calf Serum (FCS; PAA Laboratories, Austria), 50 U/ml penicillin 50 *μ*g/ml streptomycin (Sigma Aldrich) and 2 mM L-glutamine (Sigma Aldrich). NIH swiss 3T3 cells (DSMZ, Germany) were grown in DMEM supplemented with 10% FBS, 2 mM L-glutamine, and 100 U/ml penicillin 100 *μ*g/ml streptomycin. Both the cell types were cultivated in T25 flasks at 37 °C, humidified air with 5% CO_2_. The medium was changed regularly twice a week and the cells were split before they reached confluence.

For microscopy, the SH-SY5Y cells were cultured in 35 mm *μ* dishes (ibidi) which were previously coated with 50 *μ*g/ml collagen (Corning). Cells were differentiated the day after plating by 10 *μ*M All-trans retionoic acid (RA; Sigma Aldrich). After 5 days, the cells were washed three times with DMEM and grown in serum-free DMEM supplemented with 50 ng/ml brain-derived neurotrophic factor (BDNF; Sigma Aldrich) for 7 days. After treatment, cells exhibit biochemical and morphological features similar to those of mature human neurons^18^.

NIH-3T3 cells were cultured in 35 mm imaging dishes pre-coated with 10 *μ*g/ml fibronectin (Sigma Aldrich) for microscopy. Both the cell lines were liposome-transfected by pGFP-EB1 plasmid^28^ (Addgene 17234) by means of the torpedo DNA transfection vector (Ibidi) in DMEM media without sera and antibiotics according to manufacturer’s protocol. Live cell microscopy started 18–48 hrs after transfection.

### Live cell imaging and processing

Cells successfully expressing GFP were chosen and analyzed with an Axio observer Z1 inverted fluorescence microscope equipped with an Axiocam Mrm, Incuabator X1multi S1, TempModul S1, and CO_2_ Modul S1 (all from Zeiss). Images were taken every 1s at an exposure time of 600–800 ms with a 488 nm laser for 15–20 min with 100× objective. All the measurements were performed in a humidified atmosphere at 37 °C and at 5% CO_2_. Quantitative analysis of the microtubule dynamics was carried out on time-lapse movies of cells expressing EB1-GFP. Microtubule growth rates were obtained by tracking EB1-GFP comets at microtubule plus-ends. Images were recorded and movies were assembled by means of AxioVision software. More than 750 MT tips in Fibroblast cells and nearly 800 MT tips in axons of human neurons were analyzed.

## Results

### Characterization of MT dynamics in fibroblast cells

We first study fibroblast cells, in which the dynamic behavior of MTs is essential for cytoskeletal reorganization. We measure the phenomenological characteristics of MT dynamics such as the growth velocity and the frequencies of rescue or catastrophe events, and clarify their differences in the cell interior compared to the cell margin. In the model section we demonstrate how these differences correspond to different MT growth strategies and lead to distinct steady-state MT lengths.

**Figure 1:**
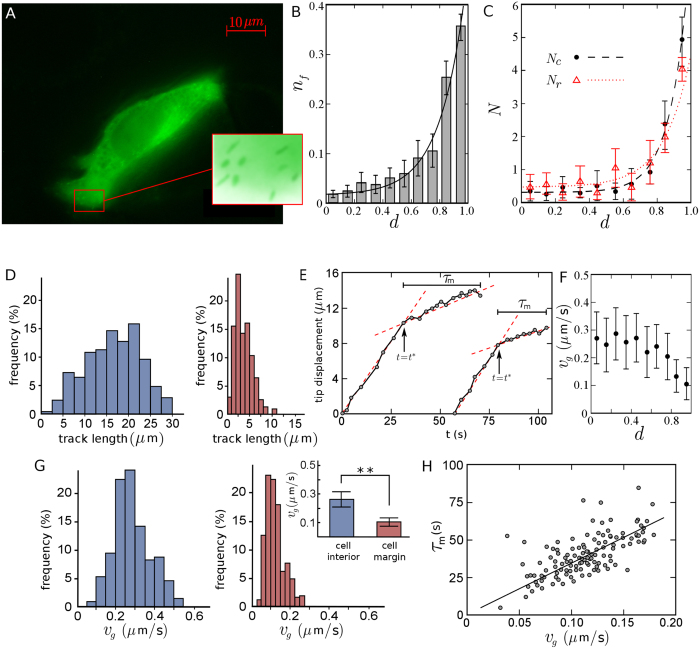
MT dynamics in fibroblast cells. (**A**) Distribution of EB1 labeled microtubule tips (spots) in a fibroblast cell. The inset shows a zoomed part of the cell margin. (**B**) The fraction *n*_*f*_ of active plus ends versus *d*, the relative position of MT tip between the cell interior and margin. The solid line indicates the best fit to an exponential growth 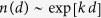 with *k* = 4.36 ± 0.37. The imaging frequency is 1 frame/s and the total observation time is 850 s. (**C**) The number of rescue *N*_*r*_ and catastrophe *N*_c_ events (per 100 growing tips) versus *d*. The dashed and dotted lines denote exponential fits with constants 9.06 ± 0.66 and 6.81 ± 1.61, respectively. (**D**) Frequency histogram of the excursion length of polymerization (i.e. the run length in the growth phase) in the cell interior (*d* < 0.8, left) and near the cell margin (*d* > 0.8, right). (**E**) Temporal evolution of the position of a few typical plus-end tips that reach the cell margin. The dashed lines are obtained from the three-parameter fit, as explained in the text. (**F**) The growth velocity v_*g*_ versus *d*. (**G**) Frequency histogram of the growth velocity in the cell interior (left) and near the cell margin (right). The inset shows the corresponding mean values (*P* ≤ 0.01, t test). (**H**) Scattered data points showing the excursion time of polymerization near the cell margin, *τ*_*m*_, versus the growth velocity, v_*g*_. The solid line indicates the best linear fit.

The distance of MT tip from the cell margin is a decisive parameter in determining its dynamics. To investigate how MT dynamics changes when approaching the fibroblast cell margin, we characterize the location of each MT plus-end with respect to the centrosome and plasma membrane by a dimensionless quantity *d* ranging from 0 (center) to 1 (margin). In case of elongated fibroblast cells, *d* denotes the relative distance of the MT tip to the plasma membrane parallel to the direction of elongation of the cell. Otherwise, *d* is calculated for each individual MT tip based on its current position (i.e. in the chosen image frame) along its trajectory. We checked that other choices to characterize the location of the MT tip with respect to the cell margin, such as the absolute distance from the membrane, lead to qualitatively similar results for the behavior of MT dynamics parameters as a function of the location of the MT tip. The analysis of live-cell images reveals that the plus ends are more concentrated in the vicinity of the cell margin (see Fig. 1A,B). The increase of the fraction of active tips *n*_*f*_ with the relative distance *d* is accelerated towards the plasma membrane, which can be quantitatively described by an exponential growth in *d*. The question arises how the cell manages to adjust the spatial distribution of the MT tips. To address this, we measure the accessible quantities related to the dynamics of MTs and consider their evolution as a function of the distance from the membrane, which provides a better understanding of the underlying mechanisms of MT length regulation.

The relatively high density of tips near the membrane is a signature of unstable dynamics and more frequent switching between the growth and shrinkage phases, compared to the cell interior. We directly examine this by measuring the number of catastrophe or rescue transitions, i.e. switching events from growth to shrinkage phase and vice versa, respectively. In order to reduce the image analysis errors, a minimum life-time threshold of two successive frames after creation (before disappearance) is imposed on a signal to consider the event as a rescue (catastrophe) transition. The number of rescue *N*_*r*_ and catastrophe *N*_*c*_ events per each 100 growing tips are shown in Fig. 1C. Both *N*_*r*_ and *N*_*c*_ increase towards the cell membrane which can be described by exponential functions in terms of *d*, where the increase of catastrophe events is more pronounced. Thus, with approaching the membrane, MTs more frequently experience switching events, which prevents long excursions of polymerization/depolymerization and leads to a high concentration of active tips in the vicinity of the plasma membrane. Figure 1D shows that the probability distribution of the excursion length of polymerization 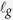 is broader in the cell interior and its average value is larger. When separating the cell interior and margin with the threshold value *d* = 0.8 (see below), we obtain 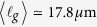 and 4.2 *μ*m, respectively, for the cell interior and margin.

We also identify individual MTs and follow their trajectories to see how the tip proceeds when it approaches the membrane. In Fig. 1E, tip displacements of a few typical MTs are shown. It can be seen that the growth velocity remains nearly unchanged in the cell interior (provided that it does not face large obstacles which are spatially constrained), but it is drastically reduced near the plasma membrane. By measuring the instantaneous growth velocity *v*_*g*_ as a function of the dimensionless quantity *d*, it is shown in Fig. 1F that *v*_*g*_ is considerably lower near the membrane. The sharp change of the growth velocity for individual MTs provides the opportunity to quantitatively discriminate between the cell interior and margin. To this aim, we fit the time evolution of the position of the tip with the following function


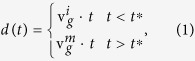


where the three free parameters 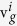, 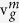, and t^*^ respectively denote the mean growth velocity in cell interior, mean growth velocity in cell margin, and the onset of transition from interior to margin. By varying t^*^ we minimize the fitting errors and obtain the best set of fit parameters (see Fig. 1E). Repeating this procedure for more than 300 MT tips, the frequency histogram of the growth velocity is separately obtained for the cell interior and margin (see Fig. 1G). The corresponding average values are 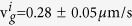 and 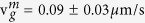. Moreover, the transition point is, on average, located at *d* ≈ 0.77. We also measure the excursion time τ_m_ of polymerization near the cell margin. The resulting values are plotted in terms of 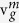 in Fig. 1H. Interestingly, we observe a positive correlation between these quantities (their Pearson correlation coefficient is nearly 0.57); the faster the MT tip is, the longer it survives in the growing phase.

### MT dynamics in axons of human neurons

**Figure 2:**
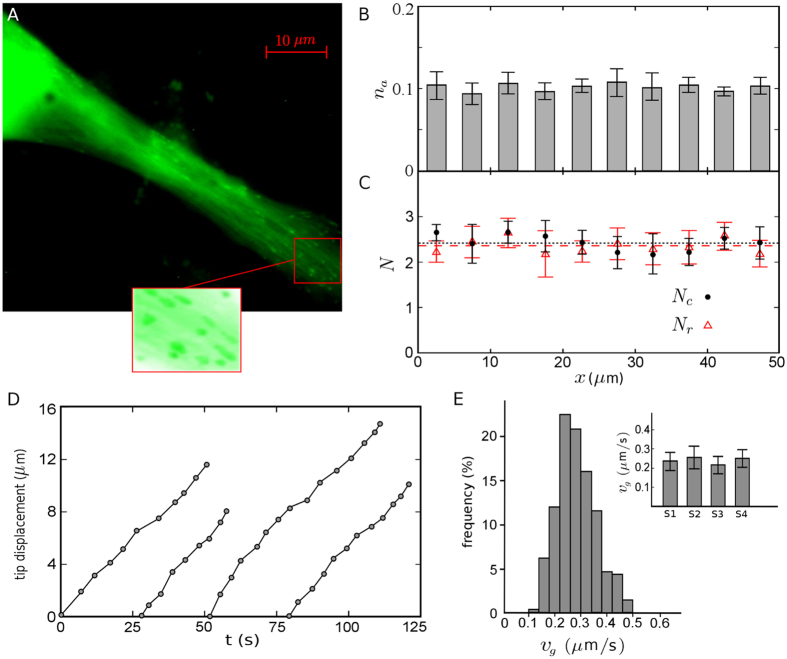
MT dynamics in axons of human neurons. (**A**) Distribution of EB1 labeled microtubule tips (spots) in axons. The inset shows a zoomed part of the axon. (**B**) The linear density *n*_*a*_ of active plus ends (corrected for the variations of the cross-section area of axon) versus the distance *x* from the soma. A distance of 50 *μ*m is considered. The imaging frequency is 1 frame/s and the total observation time is 850 s. (**C**) The number of rescue *N*_*r*_ and catastrophe *N*_*c*_ events (per 100 growing tips) versus the distance *x* from the soma. The dashed lines indicate mean values. (**D**) Temporal evolution of the position of a few plus-end tips along the axon. (**E**) Frequency histogram of the growth velocity *v*_g_. Inset: The mean value of v_*g*_ obtained from four different axons.

Next, we investigate MT dynamics in axons of neurons. In the absence of MT organizing center, segments of MTs are distributed nearly parallel to the plasma membrane. As MT tips do not contribute in keeping the shape of axons, it is expected that the parameters describing the dynamic structure of MTs are rather homogeneously distributed. Taking the variations of the thickness of axon tubes into account, we find that the linear density of active tips is uniform along the axon (see Fig. 2B), evidencing that the distance from the soma is not an influential parameter for MT dynamics. Our detailed analysis also showed no significant difference in the density of plus-end tips across the cross-section of axon (not shown). Additionally, it can be seen from Fig. 2C that the number of rescue and catastrophe events remain invariant along the axon. Their average values are smaller than those of the margin of fibroblast cells but greater than the fibroblast cell interior. In the next section we analytically verify how these differences lead to piecewise MT segments of various lengths in axons versus a persistent MT growth in bulk and a stable cytoskeleton in fibroblast cells.

**Figure 3:**
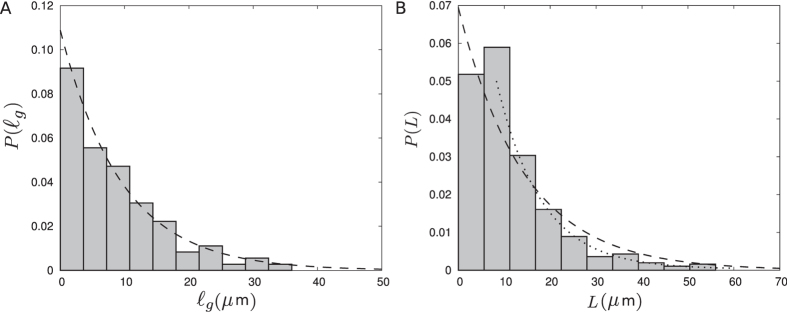
(**A**) The probability distribution of the length 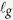 of polymerization episodes. The dashed line is an exponential fit 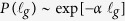 with α = 0.11 ± 0.01 *μ*m^−1^. (**B**) The estimated probability distribution of the MT length *L*. The dashed line corresponds to the analytical prediction via Eq. (4) and the dotted line is an exponential fit to the tail.

By tracing individual MT trajectories we observe that the tips do not experience significant changes in their growth speed until the catastrophe occurs (see Fig. 2D). Thus, the behavior is different from the MT dynamics near the cell margin of fibroblast cells. The quick returns to the growth phase due to high rescue rate do not exist here, and the shrinkage periods are more persistent. The histogram of the growth velocity, shown in Fig. 2E, is qualitatively similar to that of the interior of fibroblast cells. The average growth velocity is nearly the same in all samples of axon, yielding an overall mean value of *v*_*g*_ = 0.24 ± 0.05 *μ*m/s. The probability distribution *p*(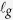) of the length of growth episodes has a mean value of 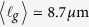 with a decaying tail which can be roughly fitted to an exponential curve, as shown in Fig. 3A.

The total length *L* of MT and its probability distribution *P*(*L*) are the quantities of interest, which can not be directly deduced from our experimental results, as the labeling only visualizes the plus ends in the growth state and the positions of the minus ends are unknown. However, we can indirectly extract an approximate value of the average MT length 〈*L*〉 and the shape of the length distribution, by estimating the capacity of the axon tube from the analysis of MT trajectories. Denoting the mean number of MTs accommodated in the cross-section of the axon by *q*, we start from an arbitrary imaginary cross-section and measure the distance *x* along the axon to reach the *q*-th plus-end tip in a given image frame. The average value of the fluctuating quantity *x* corresponds to the half of the typical MT length. Thus, by moving the reference cross-section along the axon, correcting the data for the variations of the axon thickness, and repeating the procedure for all of the image frames, we obtain the average MT length 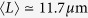. Moreover, we can construct the probability distribution *P*(*x*), which is a cumulative distribution function from which *P*(*L*) can be deduced. The resulting probability distribution *P*(*L*) shown in Fig. 3B develops a small peak at short lengths and has a fast decaying tail, indicating that MTs with a length much longer that the average value are highly improbable.

## Model

The MT length regulation mechanism has been theoretically studied over the last two decades^9^,^10^,^29^,^30^,^31^,^32^. More recently, the interplay between polymerization kinetics and motor-induced depolymerization has been incorporated into the stochastic models for the length regulation of MTs and other active biopolymers^9^,^10^. However, to predict the filament length via these models requires detailed information such as the motor concentration on the filament, which is experimentally inaccessible. Instead, a conceptually more simple model, proposed by Dogterom and Leibler^32^, builds on a few parameters that can be more easily measured. In this phenomenological model, the evolution of the MT length is described in terms of its growth v_g_ and shrinkage v_s_ velocities and the frequencies of catastrophe f_c_ and rescue f_r_ events. Here we follow such an approach to obtain the average steady-state length and its probability distribution as well as the conditions under which the filament length diverges.

We introduce *p*_*g*_ (*L*, *t*) and *p*_*s*_ (*L*, *t*) as the probabilities of having a filament of length *L* at time *t* being in the growth or shrinkage phase, respectively. One can describe the evolution of these probabilities by the following coupled master equations


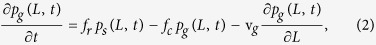



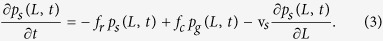


While these equations can be analytically solved in general by considering appropriate boundary conditions to obtain the time evolution of MT length, the steady-state behavior is of the main interest. Therefore, by setting the left hand sides of the above equations to zero, after some calculations one obtains the probability distribution of the filament length in the steady-state as


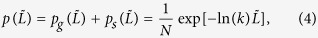


where 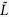 denotes the dimensionless length of MT in units of tubulin dimer length 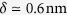 (i.e. 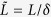), *N* is the normalization factor, and 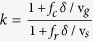. The average MT length at the steady-state follows


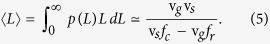


Thus, the steady-state length distribution is governed by the phenomenological parameters: the growth and shrinkage rates and the frequencies of catastrophe and rescue events. Among the four parameters of the model, *f*_*c*_, *f*_*r*_, and v_*g*_ can be directly extracted from the analysis of the live cell images. The catastrophe frequency is defined as the number *N*_*c*_ of growth to shrinkage transition events (i.e. the number of vanishing MTs between two frames) over the integrated time *T*_*g*_ spent by MTs in the growth phase. Let us assume for simplicity that the imaging frequency is 1 frame/s. Then, *T*_*g*_ equals to the number *N*_*g*_ of growing MT tips in the first frame, and one obtains


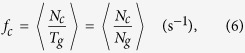


where 〈…〉 denotes averaging over all frames. Both *N*_*c*_ and *N*_*g*_ can be obtained from the image analysis, which enables us to straightforwardly evaluate *f*_*c*_. One can similarly define the rescue frequency as


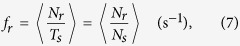


with *N*_*r*_, *T*_*s*_, and *N*_*s*_ being the number of shrinkage to growth transition events (i.e. new MTs), the integrated time spent by MTs in the shrinkage phase, and the total number of shrinking MT tips in the first frame, respectively. However, *N*_*s*_ is not directly accessible since EB1 labeling technique only visualizes the dynamics in the growth phase. From 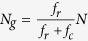 and 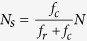 (*N* denotes the total number of MTs in a frame), we get 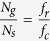. From this relation and Eq. (7), we obtain *f*_*r*_ by a two-parameter fit. The results are given in Table 1. For the remaining parameter, i.e. the shrinkage rate v_*s*_, we take v_*s*_ ∼ 0.59 ± 0.21*μ*m/s from the literature^33^, as we do not expect that v_*s*_ is considerably affected by the presence of obstacles or in the vicinity of the cell margin. From Eq. (5) one obtains 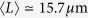 in a good agreement with the experimental value 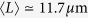, despite all the approximations made in the model as well as the inaccuracies in evaluating the quantities of interest in experiments. We also compare the analytical prediction of *p* (*L*) from Eq. (4) with the distribution estimated from the experimental data. As shown in Fig. 3B, there is a remarkable agreement, even though the small peak is not captured.

## Discussion

We investigated the altering phases of polymeriztion and depolymerization of MTs in different cell types and regions, and showed that the dynamics of MTs in fibroblast cells is distinctive from neuronal axons. The behavior even differs considerably between the interior and margin of fibroblast cells, i.e. after reaching from the cell interior all the way to the plasma membrane, the growth dynamics of MTs significantly changes. Importantly, MT tip fluctuations and collecting more active tips near the membrane enables the cell to quickly respond to changes in the environmental conditions, adapt its shape, or advance its edge and move. In contrast, the tip experiences a persistent growth/shrinkage phase within the cell interior, which results in a rather stable filamentous structure in the bulk of the cell. The combination of the two types of MT dynamics in bulk and margin allows the cell to maintain the connection between nucleus and membrane.

The growth velocity of MT tips in our experiments, even in the margin of fibroblast cells, is far greater than those obtained from in vitro experiments of MT growth against rigid obstacles^34^,^35^,^36^ or those with coated beads coupled to their ends^37^, thus, these conditions were different and we cannot reasonably consider the force-speed relations that were determined in these works for interpretation of our observations. The persistent intracellular growth followed by a highly unstable dynamics near the cell margin was also reported in other eukaryotic cells, such as CHO-K1 cells^33^, which was attributed to the promotion of rescue rate near the membrane induced e.g. by the presence of CLIP-170 linker proteins^33^. These proteins also enable MTs to distinguish different cortical regions and regulates their catastrophe rate accordingly^38^. Moreover, it has been shown that barrier-attached dynein can inhibit MT growth and trigger microtubule catastrophes^39^. The lack of GTP in the vicinity of the membrane, and also the spatial variations of the distribution of mitochondria can be other influential factors to the dynamics of MTs at different cell regions. Understanding the underlying mechanisms of MT dynamics regulation is crucial and requires further detailed studies, which is the subject of our ongoing research.

**Figure 4:**
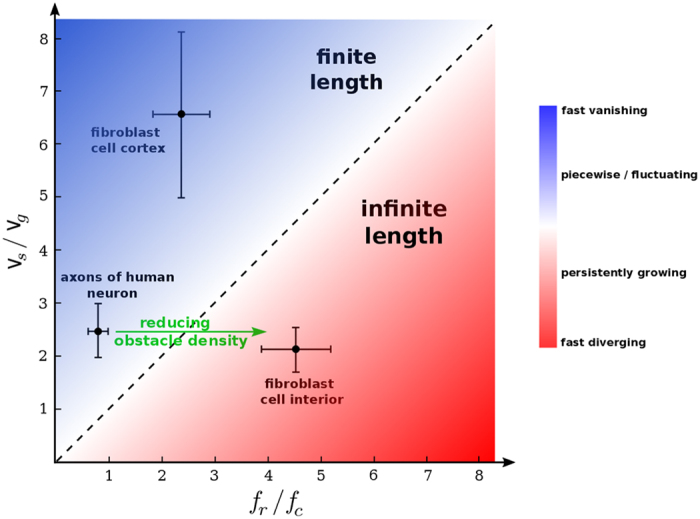
Phase diagram of MT length regulation in the v_*s*_/v_*g*_ − *f*_*r*_/*f*_*c*_ plane. The dashed line indicates unity and separates the steady-state diverging and finite length regimes. The color intensity indicates possible scenarios of MT dynamics.

From the phenomenological model of MT length regulation one obtains a criterion for the transition from finite length to diverging MTs. It can be deduced e.g. from Eq. (5) that the MT length diverges if 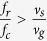. Therefore, we summarize the MT length regulation in a phase diagram in the ν_*s*_/ν_*g*_ − *f*_*r*_/*f*_*c*_ plane in Fig. 4. While the parameter values corresponding to the interior of fibroblast cells are located in the diverging regime of the phase diagram, the model successfully predicts a finite steady-state length for both the fibroblast cortex and the axons of neurons. A comparison between axon and fibroblast cell interior reveals that the parameter values are very similar, except for the catastrophe frequency *f*_*c*_ which shows a sixfold increase in axons. A plausible scenario is that MT parameter values in axons are designed for an infinite growth along the tube, similar to the bulk of eukaryotic cells. However, the frequency of transition from growth to shrinkage phase increases when growing against large obstacles in laterally-limited crowded tubes of axons, which causes a piecewise MT segment structure. Thus, it is expected that *f*_*c*_ decreases and MTs grow unlimitedly with reducing the obstacle density in the axon tube. Further investigations of the dynamics and spatial organization of MTs in axons remain for future work, which is a crucial step towards a better understanding of the underlying mechanisms of bidirectional transport driven by cytoskeletal motors^40^ and the impact of neurodegenerative disorders on it. More generally, the efficiency of the intracellular transport substantially depends on the structure of the cytoskeleton^41^,^42^, which underlines the need of detailed studies in other cell types to understand the structural dynamics of cytoskeletal biopolymers and their spatial variations with respect to the cell boundaries. As a final remark, we observed no aging effects in MT dynamics in the biological systems under consideration within the temporal resolution of our experiments, which allowed us to adopt a simple set of master equations (2) and (3) to describe the MT dynamics. In general, however, the MT dynamics can be age and length dependent^43^,^44^. In such cases, the formalism can be generalized by explicitly including the time and length dependence of the phenomenological parameters. It is also possible to analytically handle the spatial and temporal variations of the free tubulin concentration which influences the MT dynamics^32^,^45^.

## Additional Information

**How to cite this article**: Shaebani, M. R. *et al*. Tracking of plus-ends reveals microtubule functional diversity in different cell types. *Sci. Rep*. **6**, 30285; doi: 10.1038/srep30285 (2016).

## Figures and Tables

**Table 1:** MT dynamics parameters.

	Catastrophe frequency *f*_*c*_ (s^−1^)	Rescue frequency *f*_*r*_ (s^−1^)	Growth speed *v*_*g*_ (*μ*m/s)
Fibroblast cell interior	0.004 ± 0.002	0.018 ± 0.004	0.28 ± 0.05
Fibroblast cell margin	0.049 ± 0.018	0.114 ± 0.027	0.09 ± 0.03
Axons of human neuron	0.024 ± 0.008	0.019 ± 0.006	0.24 ± 0.05
